# Supplementary material for: Hydrogen-bonded organic frameworks in solution enables continuous and high-crystalline membranes
Source: Nat Commun. 2024 Jan 20;15:634. doi: 10.1038/s41467-024-44921-z (PMC10799873; doi:10.1038/s41467-024-44921-z)
Supplement: Supplementary file 1 — Supplementary Information [file 41467_2024_44921_MOESM1_ESM.pdf]

Supplementary Information

Hydrogen-Bonded Organic Frameworks in Solution Enables Continuous and High-Crystalline Membranes

Qi Yin[1], Kuan Pang[1], Ya-Nan Feng, Lili Han, Ali Morsali, Xi-Ya Li, and Tian-Fu Liu[*]

[1] These authors contributed equally

* Corresponding authors:
Tian-Fu Liu: tfliu@fjirsm.ac.cn

Requests for materials should be addressed to T-F. L. Email: tfliu@fjirsm.ac.cn

This PDF file include:

Materials, Instrumentations, and characteristics
Supplementary Figure 1 to 27
Supplementary Table 1 to 3
References

Content

Supplementary Section 3
Ligand synthesis[1-3] 4
Synthesis of powdery HOF materials 5
Synthesis of PFC-1[2] 5
Synthesis of HOF-BTB[4] 5
Synthesis of PFC-72-Co[3] 6
Preparation of HOF casting AAO membrane 6
Preparation of HOF-BTB@AAO membrane 6
Preparation of BTB@AAO membrane 6
Preparation of PFC-1@AAO membrane 6
Preparation of TBAPy@AAO membrane 7
Preparation of HOF membrane with different substrate 7
Preparation of HOF-BTB@HBG membrane 7
Preparation of HOF-BTB@ITO membrane 7
Preparation of HOF-BTB@PET-ITO membrane 7
Preparation of HOF-BTB@Cu membrane 8
Preparation of HOF-BTB@AAO_DMA membrane 8
Preparation of PFC-72-Co@AAO membrane 8
Gas permeance and selective separation 9
Gas sorption isotherms 9
Heat of sorption 10
Activation enthalpy of permeation 10
Activation enthalpy of diffusion 11
Cryo-Transmission electron microscopy 11
Three-dimensional-Electron Diffraction (3D-ED) 11
Supplementary Figures 12
References 23

Supplementary Information

Supplementary Section

Unless otherwise mentioned, all reagents and solvents were commercially purchased and used as received without further purification. 1,3,5-Tris(4-carboxyphenyl)benzene (BTB) was purchased from Energy Chemical Co. Ltd without further purification. ^{1}H-NMR spectra were recorded on Bruker AVANCE III 400MHz spectrometers. Two-dimensional NOESY spectra were measured at JNM-ECZ400S (JEOL Ltd.). PXRD was performed on Rigaku Miniflex 600 Benchtop X-ray diffraction instrument. Film XRD was characterized by Rigaku SmartLab (PhotonMax high-flux 9 kW rotating anode X-ray source). The gas isotherms were measured using ASAP 2020 and ASAP 2460 from Micromeritics Co. Ltd. SEM was performed on a Phenom Desktop Scanning Electron Microscope G6 pure, Zeiss Gemini Sigma-300 Scanning Electron Microscope, or JEOL JSM6700-F/Hitachi SU8010 Field Emission Scanning Electron Microscope. Contact angles were characterized by Theta Lite apparatus from Biolin Scientific corporation. FTIR spectra were collected at VERTEX70 series FT-IR spectrometers with ATR mode. Zeta potential was collected at Brookhaven BI-200SM. Dynamic light scattering (DLS) data were collected on Brookhaven Omni (Nano Brook Omni, Brookhaven Instruments Corporation, USA). Anodic Aluminum Oxide (AAO) disks with ordered channels about 130±20 or 300±20 nm were directly bought from PUYUAN NANO Co. Ltd (the membrane diameter of 12 mm and the thickness of 105 μm). The high-boron glass (HBG) and indium tin oxide (ITO) glass were directly bought from Luoyang GULUO GLASS Co. Ltd. The indium tin oxide polyethylene terephthalate (ITO-PET) were purchased from the supplier. The Cu sheet was purchased from Sigma-Aldrich.

Ligand synthesis[1-3]

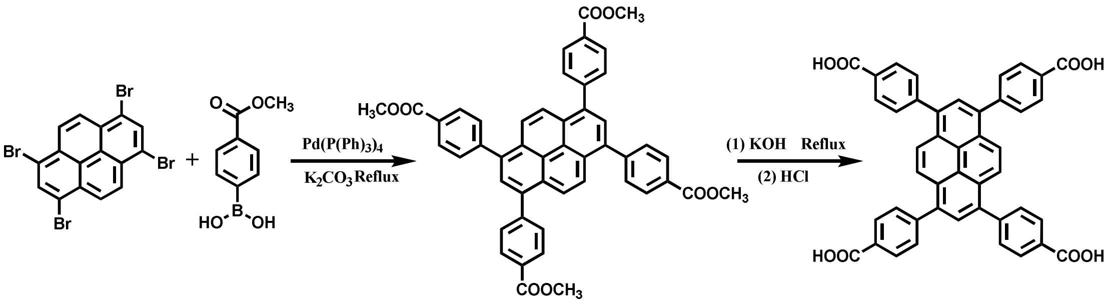

A mixture of (4-(methoxycarbonyl)phenyl)boronic acid (5 g, 32.9 mmol), 1,3,6,8-tetrabromopyrene (2.85 g, 5.5 mmol), palladium tetrakis(triphenylphosphine) (0.1 g 0.09 mmol), and potassium carbonate (6 g, 44 mmol) in dry dioxane (100 mL) was stirred under the protection of N_2 for 72 h at 85 °C. The endpoint of reaction was ascertained by thin-layer chromatography (TLC). Then the reaction mixture was poured into a mixture of ice water and concentrated hydrochloric acid (v/v = 3:1). The organic phase was extracted with chloroform and dry over Na_2SO_4. After filtration, the solvent was removed under vacuum to get crude products. Then the crude products were heated in tetrahydrofuran (THF) at 60 ^oC for 2 hours. After filtrating, the resulting solid residue was the purified product (3.21 g, yield: 78%).

Synthesis of 1,3,6,8-Tetrakis(benzoic acid)pyrene (TBAPy) Ligand

1 g (17.8 mmol, 12 equiv) KOH was added to a suspension of 1 g (1.465 mmol) 1,3,6,8-tetrakis(4-(methoxycarbonyl)phenyl)pyrene in 100 mL THF/dioxane/H_2O (v/v = 5:2:2), and the mixture was stirred under reflux at 85 °C for 12 hours. The solvent was removed under vacuum, and then 100 mL H_2O was added to the residue. The mixture (yellow clear solution) was stirred at room temperature for 2 h. The pH value was adjusted to 2 using concentrated HCl. The resultant yellow TBAPy ligand was collected by filtration, washed, and dried under vacuum (0.88 g, yield: 97%). Elemental analysis for $C_{44}H_{26}O_8$, (682.16) (%): Calcd. C 76.21 H 3.61; Found. C, 77.41; H, 3.84. ^{1}H-NMR (DMSO-d_6): δ 7.86 (d, 8H), 8.08 (s, 2H), 8.17 (d, 8H), 8.21 (s, 4H), 13.14 (s, 4H).

Synthesis of [5,10,15,20-Tetrakis(4-carboxyphenyl)porphyrinato]-Co(II) (TCPP-Co) Ligand

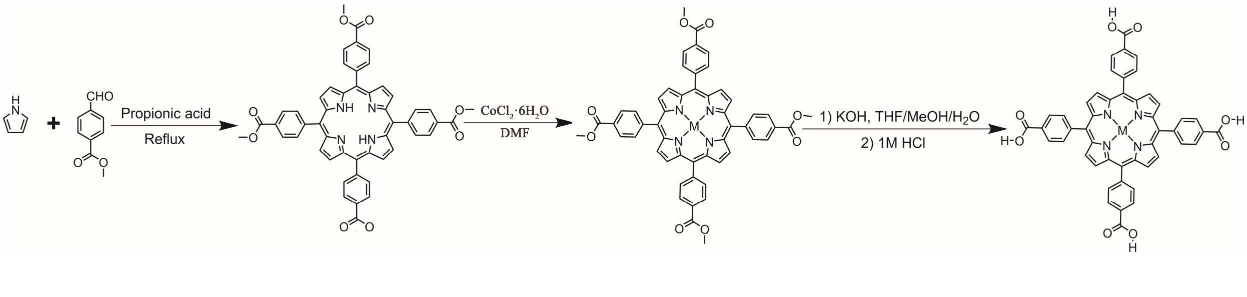

5,10,15,20-Tetrakis(4-methoxycarbonylphenyl)porphyrin (TPPCOOMe)

To refluxed propionic acid (100 mL) in a 500 mL three necked flask were added pyrrole (6.0 g, 0.086 mol) and methyl p-formylbenzoate (12 g, 0.086 mol), and the solution was refluxed for 12 h in darkness. After cooling down the reaction mixture to room temperature, crystals were collected by suction-filtration to afford purple crystals (3.6 g, 4.24 mmol, 20.2 % yield). ^{1}H NMR (300 MHz, $CDCl_3$) δ 8.81 (s, 8H), 8.43 (d, 8H), 8.28 (d, 8H), 4.11 (s, 12H), -2.83 (s, 2H).

[5,10,15,20-Tetrakis(4-methoxycarbonylphenyl)porphyrinato]-Co(II).

A solution of TPPCOOMe (0.854 g, 1.0 mmol) and $CoCl_2 \cdot 6H_2O$ (3.1 g, 12.8 mmol) in DMF (100 mL) was refluxed for 8 h. After cooling the reaction to room temperature, 150 mL of H_2O was added to the mixture. The resultant precipitate was filtered and washed with 50 mL of H_2O for two times. The obtained solid was dissolved in $CHCl_3$, followed by washing three times with water. The organic layer was dried over anhydrous magnesium sulfate and evaporated to afford equivalent amount of red powder.

[5,10,15,20-Tetrakis(4-carboxyphenyl)porphyrinato]-Co(II) (TCPP-Co)

The obtained ester (0.75 g) was stirred in THF (25 mL) and MeOH (25 mL) mixed solvent, to which a solution of KOH (2.63 g, 46.95 mmol) in H_2O (25 mL) was introduced. This mixture was refluxed for 12 h. After cooling down to room temperature, THF and MeOH were evaporated. Additional water was added to the resulting water phase until the solid was fully dissolved, then the homogeneous solution was acidified with 1M HCl until no further precipitate was detected. The red solid was collected by filtration, washed with water and dried in vacuum. FTIR (KBr): = 3423 (m), 2950 (w), 2840 (w), 1719 (s), 1605 (s), 1546(m), 1458 (m), 1394 (s), 1351 (m), 1276 (s), 1177 (w), 1112 (s), 1002 (s), 868 (w), 833 (m), 798 (s), 716 (m) cm^{-1}.

Synthesis of powdery HOF materials

Synthesis of PFC-1[2]

TBAPy (150 mg, 0.225 mmol) was dissolved in 22.5 mL DMF, to which 90 mL MeOH was added and stirred for 1 minute. The mixture stood at room temperature for 12 hours to afford yellow rodlike crystal. Then the solid was isolated by centrifugation, washed with MeOH, and soaked in acetone for 2 days. The solid was activated at 80 °C under vacuum for 10 hours (126.3 mg, Yield: 84.2%).

Synthesis of HOF-BTB[4]

Based on the modification of reported procedure[4], 200 mg BTB ligand was dissolved in 60 mL EtOH and

then heated at 90 °C for 15 min. Subsequently, the mixture was filtered to remove insoluble substance. The filtrate was unsealed and stood at RT to yield HOF-BTB by slow evaporation. The solid was isolated by centrifugation, washed with EtOH, and the soaked in EtOH for 1 day. The solid was activated at 90 ^{o}C for 10 hours (138.4 mg after one week, Yield: 69.2%).

Synthesis of PFC-72-Co[3]

TCPP-Co (20 mg, 0.025 mmol) was dissolved in 2 mL of DMF in a 15 mL uncapped glass bottle. Then 4 mL of 1,2,4-Trichlorobenzene (TCB) was added into the bottle. Then the uncapped glass bottle was hold in 100 ^{o}C oven for 2 days, then the powdery samples of PFC-72-Co were harvested.

Preparation of HOF casting AAO membrane

Preparation of HOF-BTB@AAO membrane

In general, powdery HOF-BTB was dissolved in DMF solution and sonicated for 5 s to achieve a clear casting solution. Alternatively, this solution can also be achieved by shaking the vial containing DMF and HOF-BTB for about 20 s. The sonication procedure applied here is for the purpose of rapid dissolution in DMF. AAO disk was vertically immersed in this solution for 1 minute and then transferred to a heated oven for 5 min. To achieve optimal performance, different HOF-BTB concentrations (2, 5, and 7 mg/mL), and the number of repeated casting operation (2, 5, and 10 cycles) were screened. Among which, 7 mg/mL HOF-BTB solution for 5-cycle casting operations at 100 ^{o}C gave rise to the most even and continuous HOF casting on the AAO surface (denoted HOF-BTB@AAO), and the obtained membrane was used for the followed gas separation experiment.

Preparation of BTB@AAO membrane

70 mg amorphous BTB ligand was dissolved in 10 mL DMF and then sonicated for 5 s to achieve a clear casting solution. Alternatively, this solution can also be achieved by shaking the vial containing DMF and HOF-BTB for about 20 s. The AAO disk was immersed in this solution for 1 minute and then heated at 100 °C for 5 min. After 5 cycles of repeated casting operations, BTB ligand casting membrane was obtained (denoted BTB@AAO).

Preparation of PFC-1@AAO membrane

70 mg powdery PFC-1 was dissolved in 10 mL DMF and then sonicated for 5 s to achieve a clear casting solution. Alternatively, this solution can also be achieved by shaking the vial containing DMF and HOF-BTB for about 20 s. The AAO disk was immersed in this solution for 1 minute and then heated at 100 °C for 5 min. After 5 cycles of repeated casting operations, PFC-1 casting membrane was obtained (denoted PFC-1@AAO).

Preparation of TBAPy@AAO membrane

70 mg amorphous TBAPy ligand was dissolved in 10 mL DMF and then sonicated for 5 s to achieve a clear casting solution. Alternatively, this solution can also be achieved by shaking the vial containing DMF and HOF-BTB for about 20 s. The AAO disk was immersed in this solution for 1 minute and then heated at 100 °C for 5 min. After 5 cycles of repeated casting operations, TBAPy casting membrane was obtained (denoted TBAPy@AAO).

Preparation of HOF membrane with different substrate

Preparation of HOF-BTB@HBG membrane

HBG was washed successively with suds, deionized water, and absolute ethanol, and then soaked in 50 mM NaOH solution with 30% hydrogen peroxide at 80 °C for 30 min. 2 mg powdery HOF-BTB was dissolved in 4 mL mixture solution of DMF and 1,2,4-trichlorobenzene (TCB, 1.25 µL/mL) and sonicated for 5 s to achieve a clear casting solution. Alternatively, this solution can also be achieved by shaking the vial containing DMF, TCB, and HOF-BTB for about 20 s. Subsequently, 300 µL HOF-BTB solution was casted on HBG substrate at 80 ^{o}C for 20 min, and then HBG membrane with HOF-BTB casting on the surface was obtained (denoted HOF-BTB@HBG).

Preparation of HOF-BTB@ITO membrane

ITO glass was washed successively with suds, deionized water, and absolute ethanol, and then soaked in 50 mM NaOH solution with 30% hydrogen peroxide at 80 °C for 30 min. 2 mg powdery HOF-BTB was dissolved in 4 mL mixture solution of DMF and 1,2,4-trichlorobenzene (TCB, 1.25 µL/mL) and sonicated for 5 s to achieve a clear casting solution. Alternatively, this solution can also be achieved by shaking the vial containing DMF, TCB, and HOF-BTB for about 20 s. Subsequently, 300 µL HOF-BTB solution was casted on ITO glass substrate at 80 ^{o}C for 20 min, and then ITO glass membrane with HOF-BTB casting on the surface was obtained (denoted HOF-BTB@ITO).

Preparation of HOF-BTB@PET-ITO membrane

PET-ITO was washed successively with suds, deionized water, and absolute ethanol, and then soaked in 50 mM NaOH solution with 30% hydrogen peroxide at 80 °C for 30 min. 2 mg powdery HOF-BTB was dissolved in 4 mL mixture solution of DMF and 1,2,4-trichlorobenzene (TCB, 1.25 µL/mL) and sonicated for 5 s to achieve a clear casting solution. Alternatively, this solution can also be achieved by shaking the vial containing DMF, TCB, and HOF-BTB for about 20 s. Subsequently,300 µL HOF-BTB solution was casted on PET-ITO substrate at 80 ^{o}C for 20 min, and then PET-ITO membrane with HOF-BTB casting on the surface was obtained (denoted HOF-BTB@PET-ITO).

Preparation of HOF-BTB@Cu membrane

Cu was washed successively with suds, deionized water, and absolute ethanol. The 2 mg powdery HOF-BTB was dissolved in 4 mL mixture solution of DMF and 1,2,4-trichlorobenzene (TCB, 1.25 µL/mL) and sonicated for 5 s to achieve a clear casting solution. Alternatively, this solution can also be achieved by shaking the vial containing DMF, TCB, and HOF-BTB for about 20 s. Subsequently, 300 µL HOF-BTB solution was casted on Cu sheet substrate at 80 ^{o}C for 20 min, and Cu sheet with HOF-BTB casting on the surface was obtained (denoted HOF-BTB@Cu).

Preparation of HOF-BTB@AAO_DMA membrane

20 mg HOF-BTB was dissolved in 4 mL N,N-Dimethylaniline (DMA) and then sonicated for 5 s to achieve a clear casting solution. Alternatively, this solution can also be achieved by shaking the vial containing DMF and HOF-BTB for about 20 s. The AAO disk was immersed in this solution for 1 minute and then heated at 100 °C for 5 min. After 5 cycles of repeated casting operations, HOF-BTB casting membrane was obtained (denoted HOF-BTB@AAO_DMA).

Preparation of PFC-72-Co@AAO membrane

20 mg HOF-BTB was dissolved in 4 mL DMF and then sonicated for 5 s to achieve a clear casting solution. Alternatively, this solution can also be achieved by shaking the vial containing DMF and HOF-BTB for about 20 s. The AAO disk was immersed in this solution for 1 minute and then heated at 100 °C for 5 min. After 5 cycles of repeated casting operations, HOF-BTB casting membrane was obtained (denoted PFC-72-Co@AAO).

Gas permeance and selective separation

For the single gas permeation measurement, the feed flow rate was set to 20 mL/min with Ar as sweep gas (50 mL/min). For the mixed gas permeation measurement, the gas mixture (mole ratio 1:1) was applied to the feed side of the membrane, and the feed flow rate was kept constant at 40 mL min^{-1} (20 mL min^{-1} for each gas). The pressures at both sides were kept constant at 0.1 MPa. A calibrated gas chromatograph (GC) was used to measure the concentration of single gases or mixed gases on the permeate side after the measurement system reached steady state. The average results were obtained by measuring the components three times at one pressure point. Cross-sectional SEM image shows that the thickness HOF-BTB@AAO membrane is about 105 μm.

The permeability can be calculated as follows:

$$Permeation = \frac{F_i}{L_m \cdot S \cdot P} \qquad \text{Supplementary Equation 1}$$

In which S (m^2) is the effective membrane area, P (Pa) is the pressure difference between upstream and downstream, F_i (mL/s) is the feed flow rate of gas i, L_m is the gas volume of 1 mol gas molecules under standard condition (22.4 L/mol).

The ideal gas separation factor ($ISF_{i,j}$) are calculated as follows:

$$ISF_{i,j} = \frac{P_i}{P_j} \qquad \text{Supplementary Equation 2}$$

In which i,j represent the two different permeation components, and P_x is the permeation of gas x ($x = i, j$).

For mixed gas separation, the separation factor ($SF_{i,j}$) are calculated as follows:

$$SF_{i,j} = \frac{P_i/P_j}{F_i/F_j} \qquad \text{Supplementary Equation 3}$$

In which i,j represent the two components in the mixture, and P_x, F_x ($x = i, j$) (mL/s) are the permeations in the permeate and corresponding feed flow rate of gas x, respectively.[5,6]

Gas sorption isotherms

N_2 sorption isotherms were collected on ASAP 2460 from Micromeritics Co. Ltd. at 77K to a pressure of 1 bar. Apart from N_2, the other gases were tested on ASAP 2020 from Micromeritics Co. Ltd at 298 K. The general activation procedure: as-prepared sample was allowed to soak in acetone for 48 h, during which the supernatant was replaced by fresh acetone several times to exchange and remove nonvolatile solvates. After removal of acetone by centrifugation, the samples were activated under vacuum at room temperature and then dried again in the "outgas" function of instruments at 90 °C for 10 hours prior to gas adsorption.

Heat of sorption

The heat of sorption (ΔH) of C_3H_6, and C_3H_8 for HOF-BTB were estimated from the sorption data measured at 273 and 298 K by the virial method:

$$\ln(\mathrm{P}) = \ln(\mathrm{N}) + \left(\frac{1}{T}\right)\sum_{i=0}^{m} a_i * N^i + \sum_{j=0}^{n} b_j * N^j \qquad \text{Supplementary Equation 4}$$

N: Uptake capacity (mg/g)

P: Pressure (mmHg)

T: Temperature (K)

A_i, b_j: empirical constants

R: Universal gas constant 8.314 J·mol^{-1}·K^{-1}

The heat of sorption (ΔH):

$$\text{ДH} = -R * \sum_{i=0}^{m} a_i * N^i \qquad \text{Supplementary Equation 5}$$

The use of this equation for evaluating the heat of sorption in HOFs, MOFs and activated carbons have been described previously. The Virial method is a standard method for calculating the coverage-dependent ΔH using experimental temperature-pressure-coverage isotherm data points.

Activation enthalpy of permeation

The temperature dependence of permeability also follows an Arrhenius relationship:

$$\mathrm{P} = P_0\, exp(\frac{-E_p}{RT}) \qquad \text{Supplementary Equation 6}$$

where P_0 is the pre-exponential factor, and E_p is the effective activation energy of permeation.

Furthermore, a transformed expression is shown inequation S7:

$$\ln\frac{P}{P_o} = = -\frac{E_p}{R} * \frac{1}{T} \qquad \text{Supplementary Equation 7}$$

Thus, the activation enthalpy of permeation (E_p) can be obtained by fitting permeation data at different temperatures to an expression based on Equation S8.

$$\ln\frac{P_{i1}}{P_{i2}} = = -\frac{E_p}{R} * (\frac{1}{T_{i1}} - \frac{1}{T_{i2}}) \qquad \text{Supplementary Equation 8}$$

Where P_i is the permeance (mol m^{-2}s^{-1}Pa^{-1}), T_i is operating temperature (K), and the unit of E_p is J mol^{-1}.

To facilitate the convenient calculation of E_p, which is expressed in the unit of KJ mol^{-1}, we utilize the following transformed equation, denoted as Equation S9:

$$\ln\frac{P_{i1}}{P_{i2}} = = -\frac{E_p}{R} * (\frac{1000}{T_{i1}} - \frac{1000}{T_{i2}}) \quad \text{Supplementary Equation 9}$$

Activation enthalpy of diffusion

The activation enthalpy of diffusion (E_d), the activation enthalpy of permeation (E_p) and the heat of sorption (ДH) have the relationship as the following equation S10:

$$E_p = E_d + ДH \quad \text{Supplementary Equation 10}$$

So the activation enthalpy of diffusion (E_d) can be got as following:

$$E_d = E_p - ДH \quad \text{Supplementary Equation 11}$$

Cryo-Transmission electron microscopy

To prepare samples for Cryo-TEM analysis, 20-60 drops of fresh DMF solution with 5 mg/mL HOF-BTB were dropwise added on copper-supported ultrathin carbon films (200 mesh, Zhongjingkeyi Co.), and then the film was rapidly immersed into liquid nitrogen in a temperature-controlled freezing unit (Leica EMGP, Leica, Germany), instantly allowing their vitrification. All sample preparation steps were conducted in the Leica EMGP chamber at 100 K. The HRTEM images were collected by aberration-corrected FEI Titan Themis 300 operated at 300 kV.

Three-dimensional-Electron Diffraction (3D-ED)

3D-ED data was collected by Suzhou ReadCrystal Biotechnology Co., Ltd. To prepare samples for 3D-ED analysis, 20-60 drops of fresh DMF solution with 5 mg/mL HOF-BTB were dropwise added on copper-supported ultrathin carbon films, and then the film was cooled to approximate 100 K using a liquid nitrogen cooled cryo-TEM holder before exposure to electron radiation to reduce possible damage to the particles. The continuous rotation electron diffraction (cRED) data was collected on a JEOL 2100-plus TEM equipped with MerlinEM direct electron detector under 200 kV acceleration voltage and installed with Heimdall data collection software (software developed by the ReadCrystal Tech Co.). The tilting range depended on the location of the crystals on the grid. Each frame was collected with 1 s exposure time, resulting in a 1° wedge per frame. The data was visualized with program REDp[7] and processed using XDS[8] with the aid of Coeus[9] for batch processing and merging.

Supplementary Figures

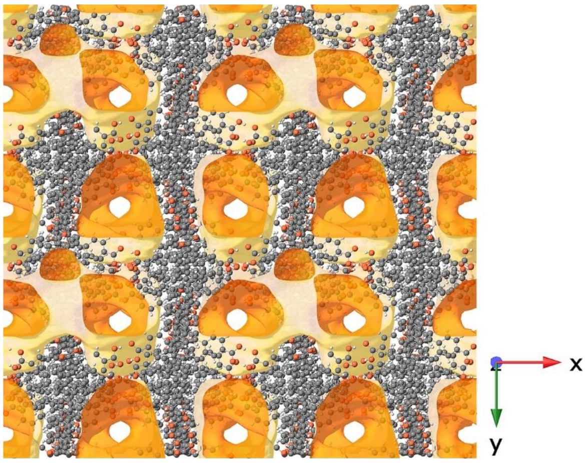

Supplementary Fig. 1. The packing structure of HOF-BTB along c axis.

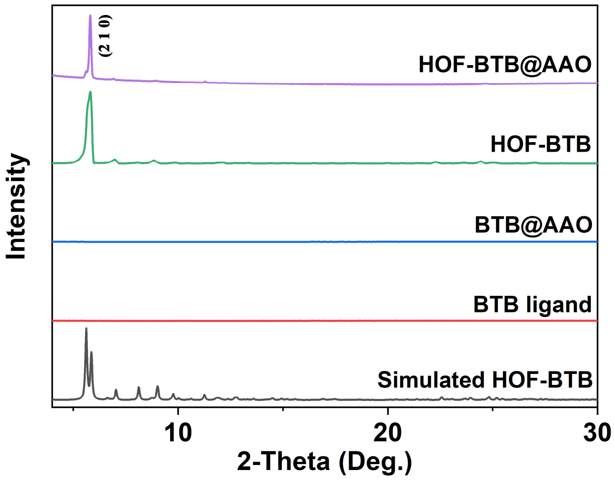

Supplementary Fig. 2. PXRD patterns of a) simulated HOF-BTB, **BTB ligand**, **BTB@AAO membrane**, as-synthesized powdery **HOF-BTB**, and **HOF-BTB@AAO membrane**.

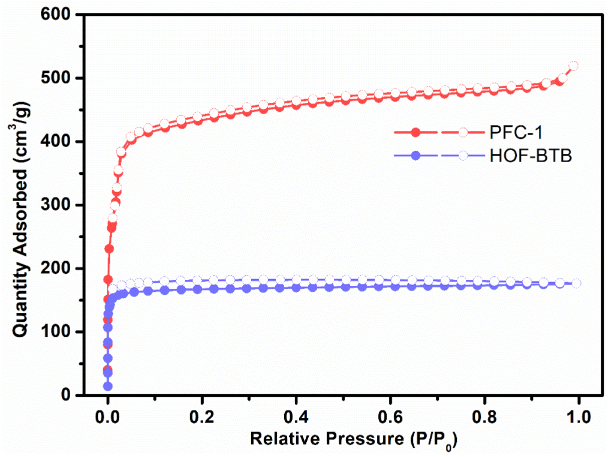

Supplementary Fig. 3. N_2 sorption isotherms of **HOF-BTB** and **PFC-1** at 77 K.

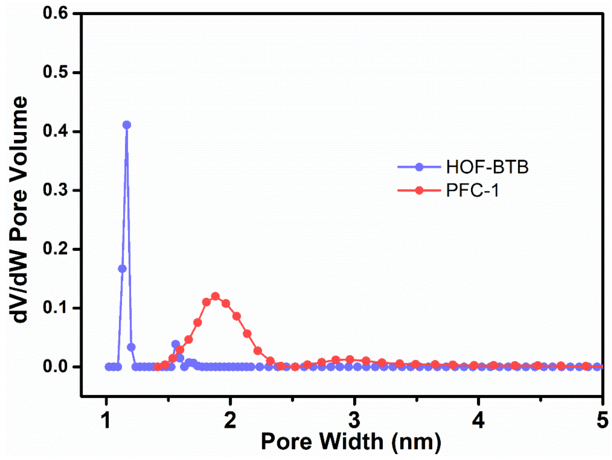

Supplementary Fig. 4. NLDFT pore size distribution of **HOF-BTB** and **PFC-1**, which matched the pore size observed by crystallographic study.

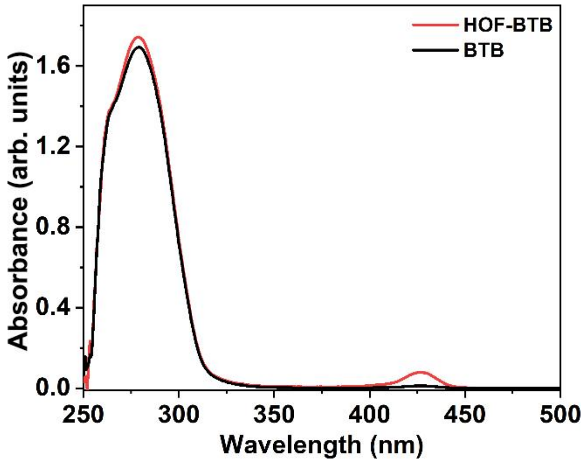

Supplementary Fig. 5. Ultraviolet–visible (UV-vis) spectroscopy spectra of **HOF-BTB** and **BTB ligand** in DMF solution, respectively.

Supplementary Table 1. zeta potentials of amorphous **BTB ligand** and **HOF-BTB**.

**Sample**	**Zeta potential**
BTB	15.65 mV
HOF-BTB	33.12 mV

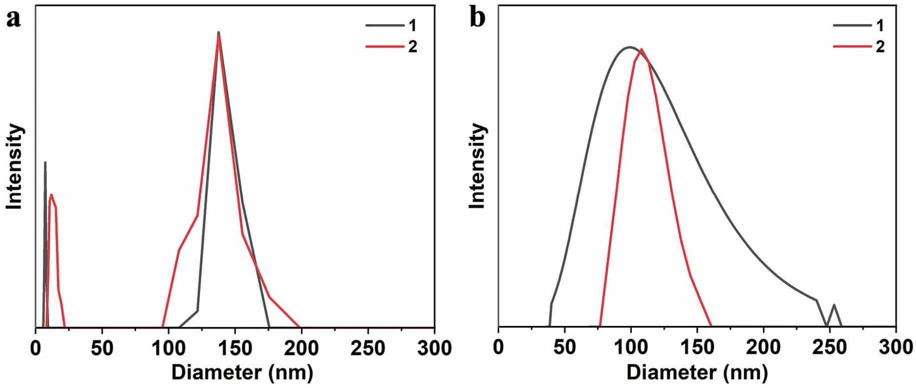

Supplementary Fig. 6. Dynamic light scattering (DLS) measurements of a) fresh **HOF-BTB** in DMF solution, and b) DMF solution of HOF-BTB after standing for one week. The experiments were repeated twice to ensure the reliability of results.

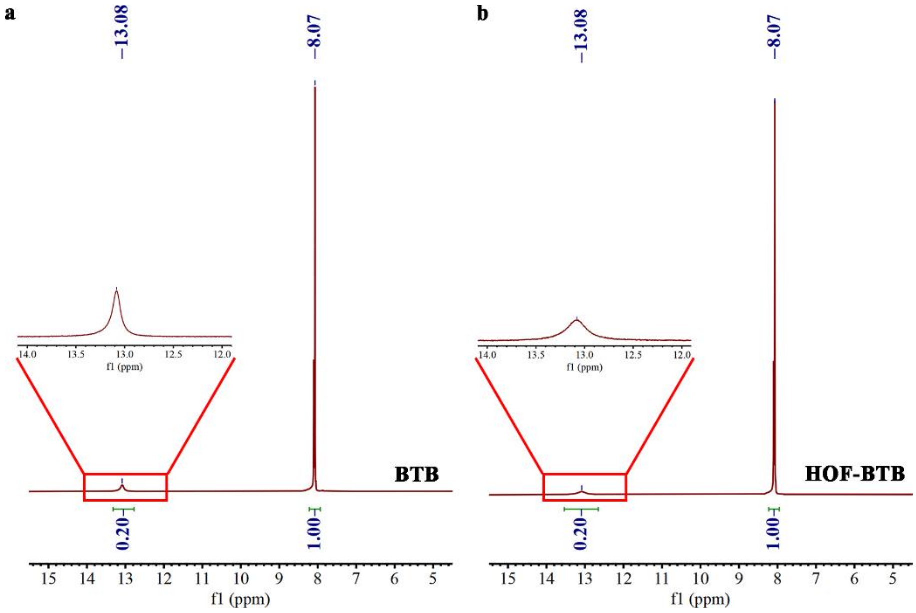

Supplementary Fig. 7. ^{1}H-NMR spectra of a) 5 mg amorphous **BTB ligand** and b) 5 mg **HOF-BTB** in 560 μL DMSO-d_6 solution.

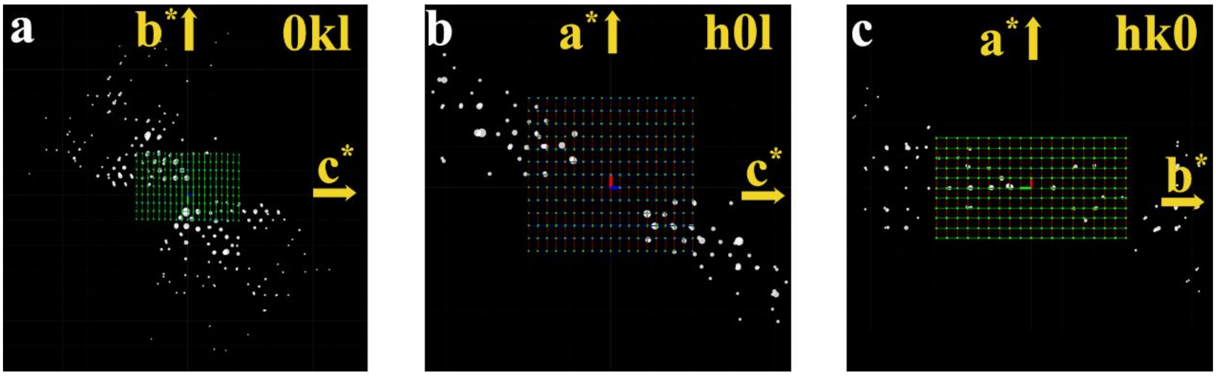

Supplementary Fig. 8. The 2D slice cut of 0kl (a), h0l (b), and hk0 (c) from the 3D reciprocal lattice. The reflection conditions deduced from the 3D reciprocal lattice: 0kl: k+l=2n; h0l: h+l=2n; hk0: h+k=2n; 0k0: k=2n, which is matched with that of I2 space group.

Supplementary Table 2. Crystallographic unit cell obtained through3D-ED and SCXRD

Data	Sample01	HOF-BTB
Source	3D-ED[a]	SCXRD[4]
CCDC number	-	1400566
a/Å	31.03	31.419(6)
b/Å	31.84	30.116(6)
c/Å	44.2	45.320(9)
α/°	90	90
β/°	90	90.412(2)
γ/°	90	90
Space group	I2[b]	I2
N_{total}	12561	96106
Completeness/%	49.0	99.8
Resolution/Å	1.20	0.77
R_{int}/%	25.25	5.96

[a]: reflection intensity extraction was conducted by the program XDS[8] with the aid of Coeus[9].

[b]: the space group was determined by the program XPrep.

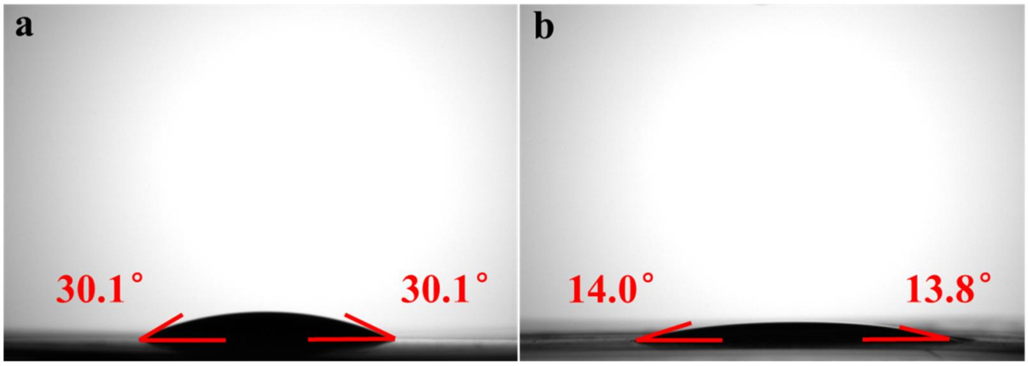

Supplementary Fig. 9. The DMF contact angles of a) dry AAO disk and b) AAO disk wetted by DMF solvent.

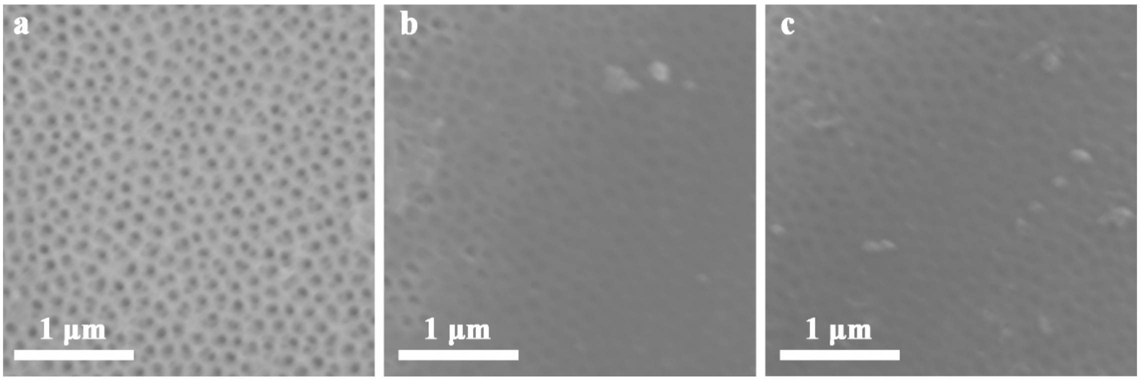

Supplementary Fig. 10. HOF-BTB membrane prepared from DMF solution of HOF-BTB with the concentration of a) **2 mg/mL**, b) **5 mg/mL**, and c) **7 mg/mL** at 100 °C for five cycle casting operation.

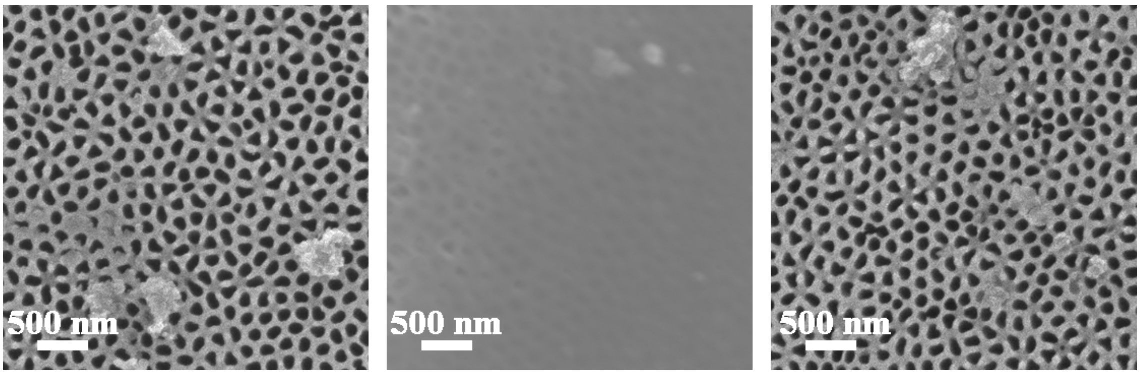

Supplementary Fig. 11. HOF-BTB@AAO membrane prepared in DMF solution with 5 mg/mL HOF-BTB at 100 °C for b) **2 cycle**, c) **5 cycle,** and d) **10 cycle** casting operation.

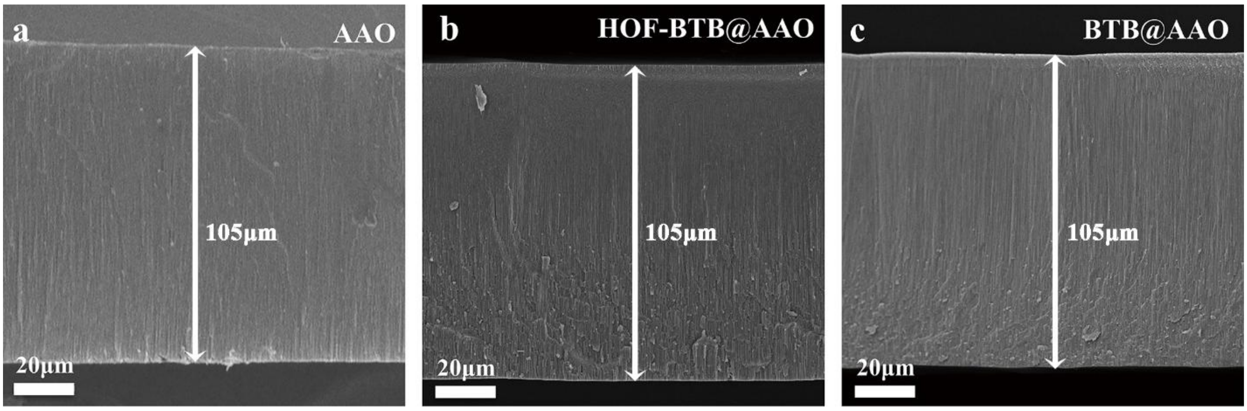

Supplementary Fig. 12. The thickness of a) AAO disk, b) HOF-BTB@AAO membrane, and c) BTB@AAO

membrane.

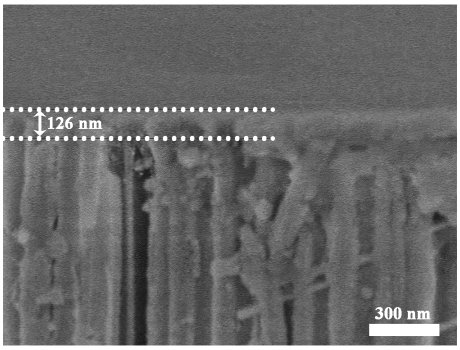

Supplementary Fig. 13. The thickness of HOF-BTB layer on HOF-BTB@AAO membrane.

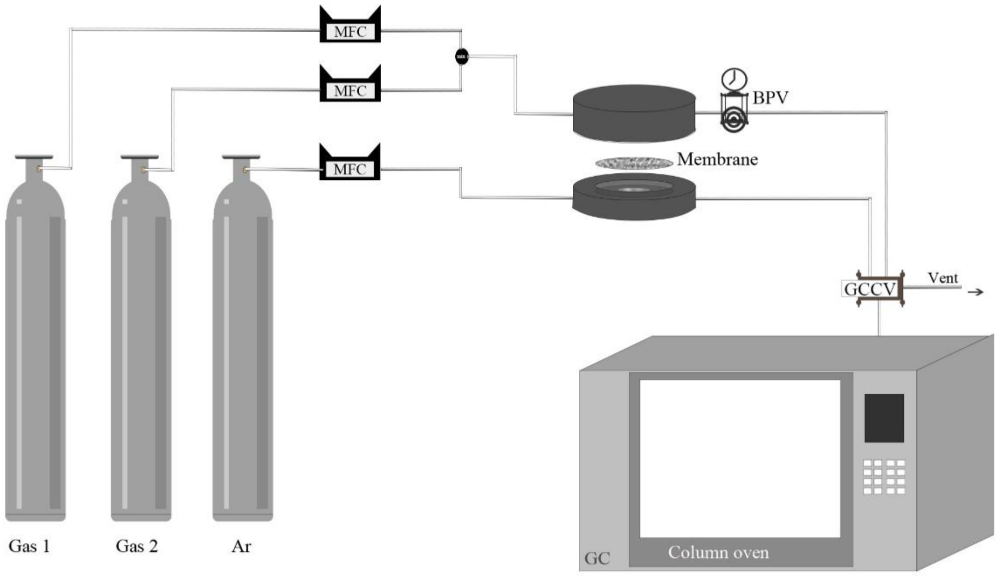

Supplementary Fig. 14. Schematic diagram of the single/binary gas permeation setup. MFC: mass flow controller, BPV: back pressure valve, GCCV: gas circuit control valve, GC: gas chromatography.

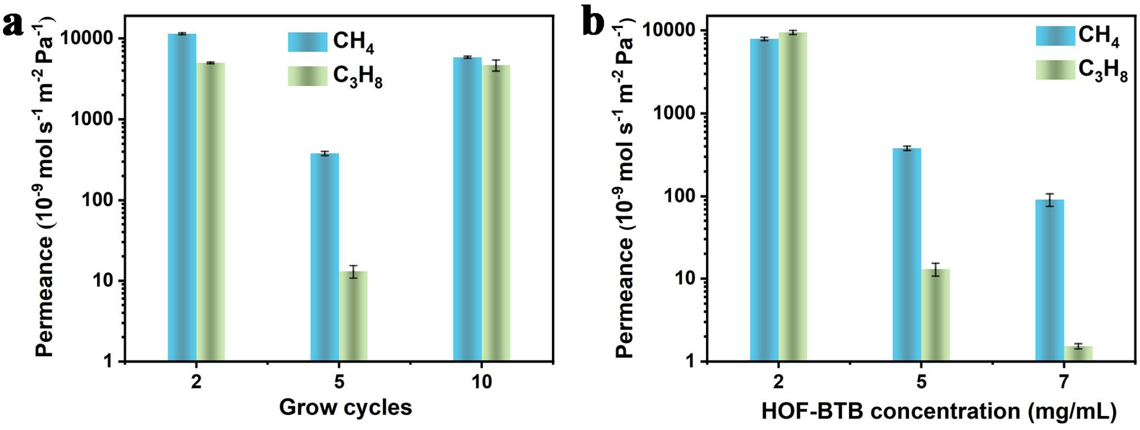

Supplementary Fig. 15. Single gas permeation of CH_4 and C_3H_8 for **HOF-BTB membranes** prepared in DMF

solution with a) 5mg/mL HOF-BTB and different cycle casting operations, and b) different HOF-BTB concentration for 5 cycle casting operations, showing that HOF-BTB@AAO membrane fabricated by 7 mg/mL HOF-BTB in DMF casting at 100 °C for 5 repeated cycles gave rise to a membrane with optimal performance of lowest C_3H_8 permeance and best CH_4 to C_3H_8 selectivity. Error bars represent standard deviations.

Supplementary Table 3. Element content analyses of AAO basement, HOF-BTB@AAO membrane, and PFC-1@AAO membrane determined by SEM-EDS.

Sample	Atomic Concentration			Weight Concentration		
	C	O	Al	C	O	Al
AAO	1.12	66.32	32.56	0.69	54.33	44.98
HOF-BTB@AAO	8.57	60.49	30.94	5.40	50.79	43.81
PFC-1@AAO	14.00	48.15	37.86	8.58	39.30	52.12

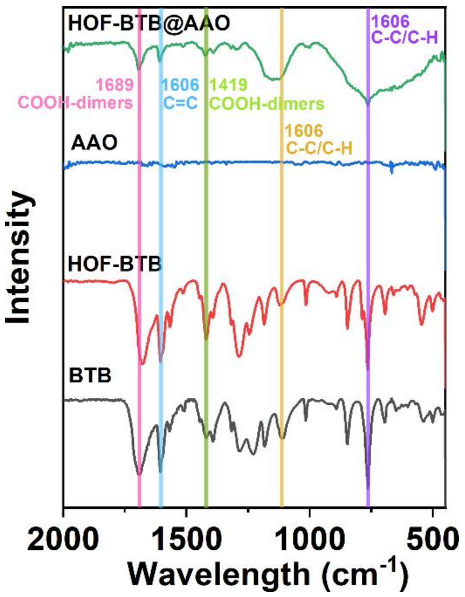

Supplementary Fig. 16. Fourier Transform Infrared (FTIR) spectra of **HOF-BTB@AAO** membrane, **AAO disk**, **HOF-BTB,** and **BTB ligand**.

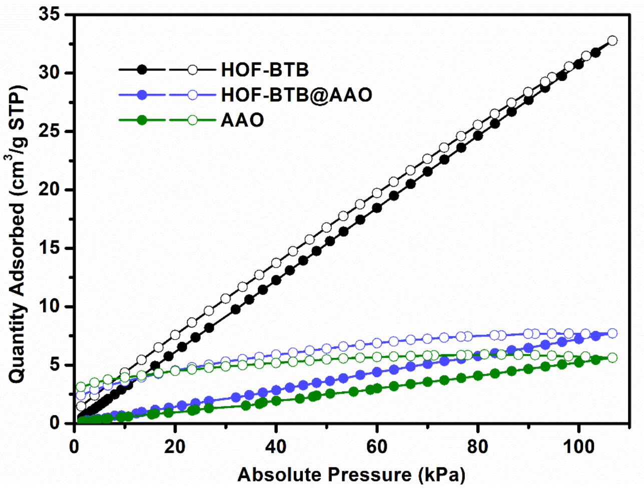

Supplementary Fig. 17. CO_2 sorption isotherms of **HOF-BTB**, **HOF-BTB@AAO membrane** and **AAO disk** at 298 K.

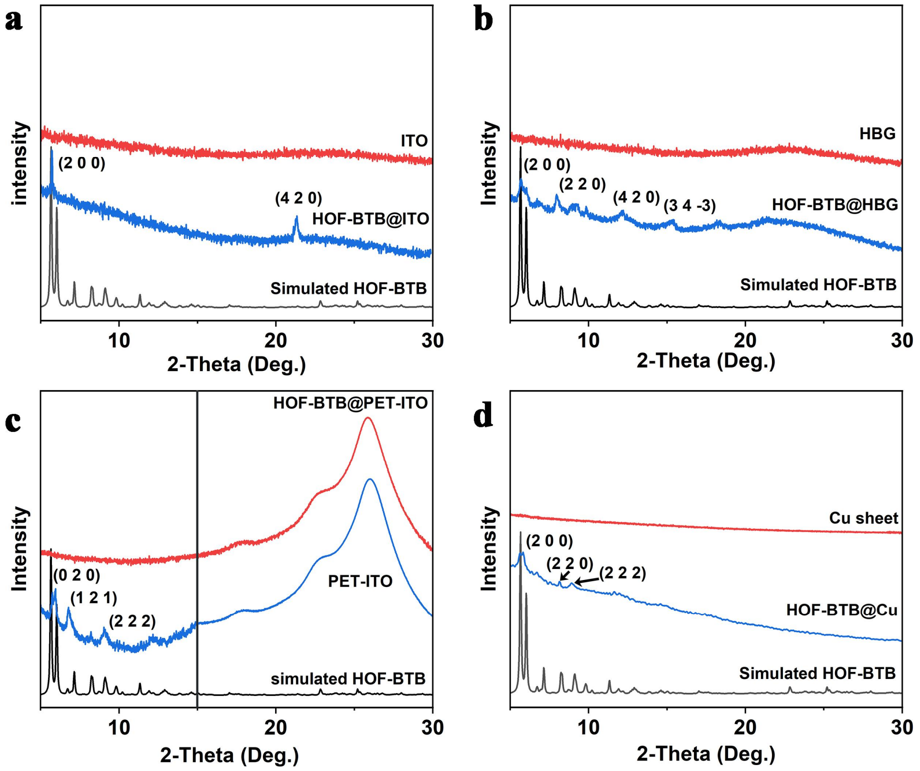

Supplementary Fig. 18. PXRD patterns of a) **simulated HOF-BTB**, **ITO glass**, and **HOF-BTB@ITO** membrane, b) **simulated HOF-BTB**, **HBG glass**, and **HOF-BTB@HBG** membrane, c) **simulated HOF-BTB**, **ITO-PET substrate**, and **HOF-BTB@ITO-PET** membrane, d) **simulated HOF-BTB**, **Cu sheet**, and **HOF-BTB@Cu** membrane.

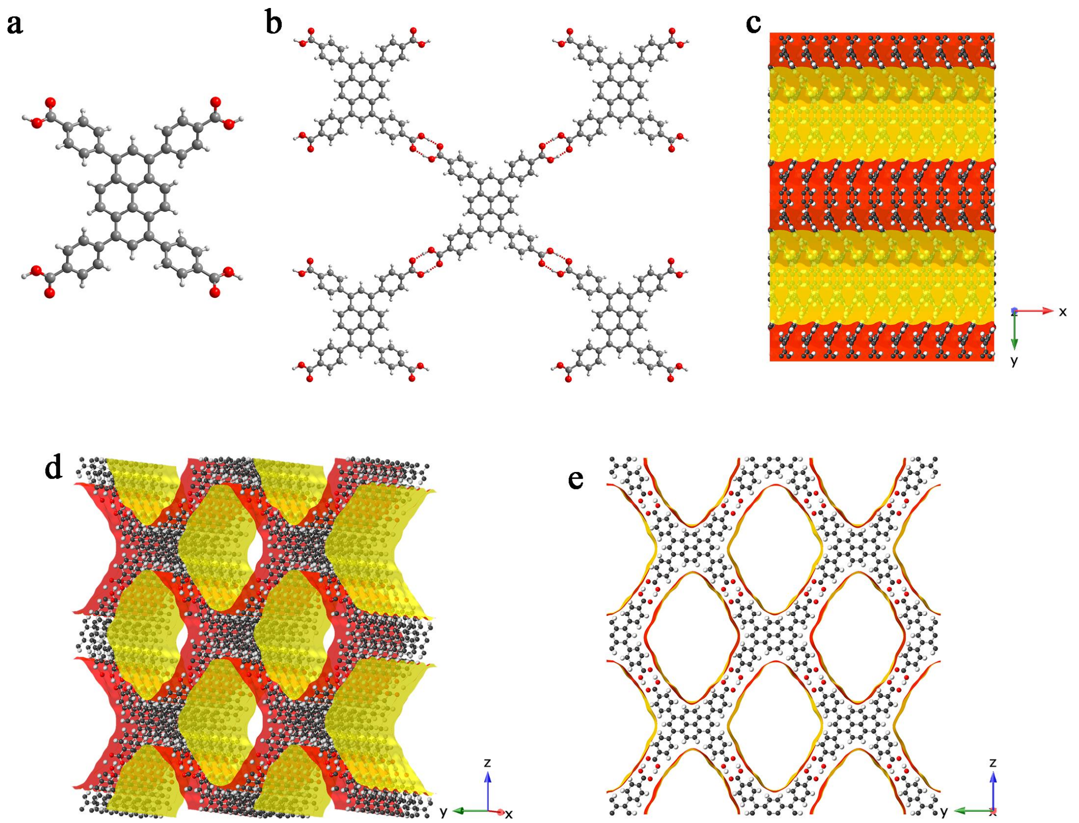

Supplementary Fig. 19. Crystal structure of **PFC-1**. a) The structure of building block; b) the connection of adjacent building blocks; c-e) The packing structure seeing from different directions with highlighting the geometry and surface of channel.

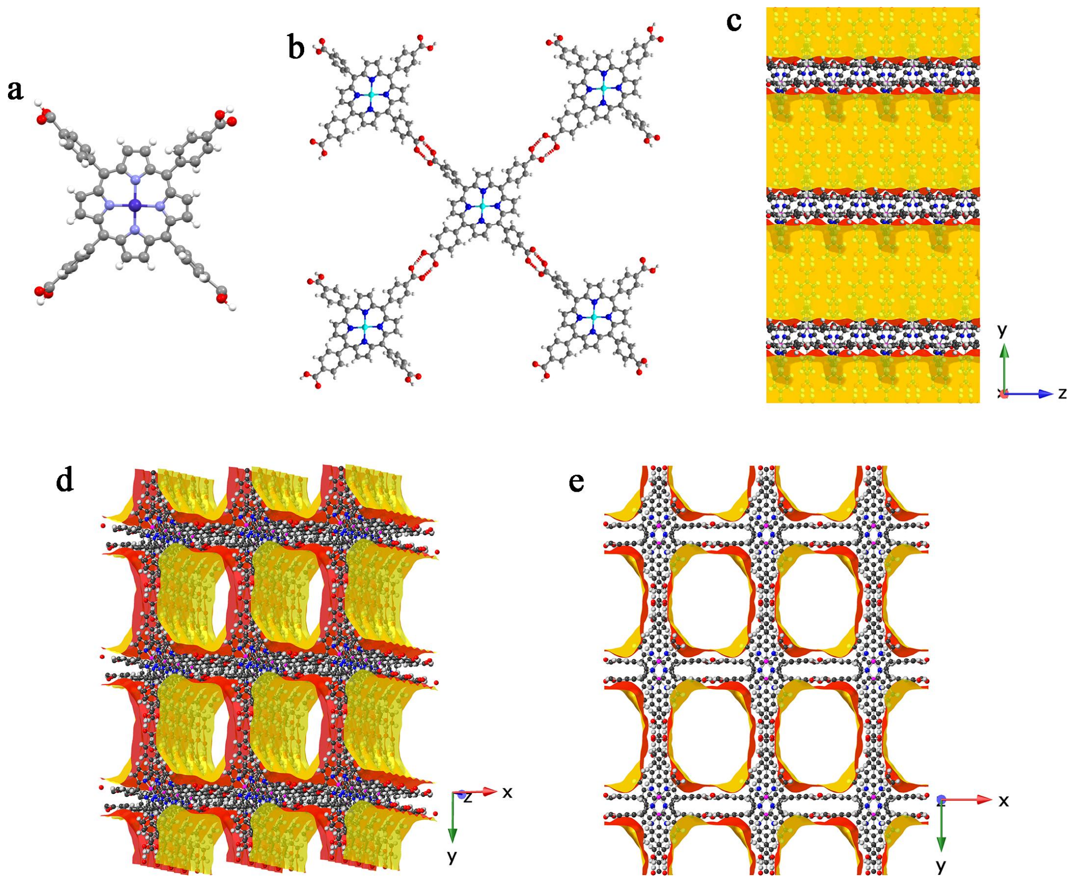

Supplementary Fig. 20. Crystal structure of PFC-72-Co. a) The structure of building block; b) the connection

of adjacent building blocks; c-e) The packing structure seeing from different directions with highlighting the geometry and surface of channel.

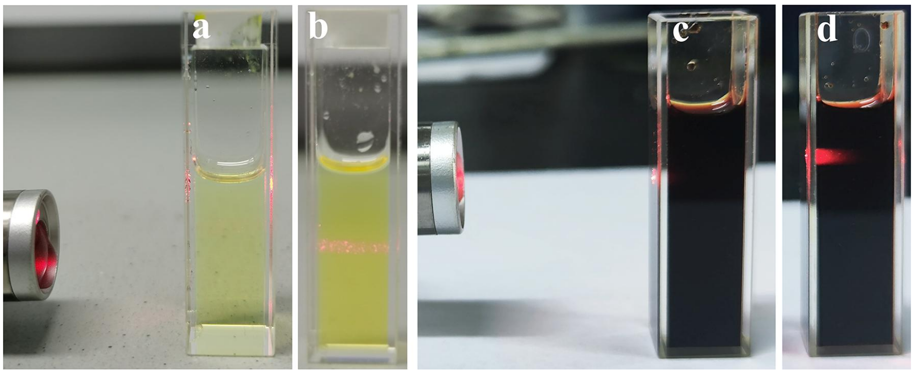

Supplementary Fig. 21. Optical photographs of DMF solution with a) 5 mg/mL amorphous **TBAPy ligands**, b) 5 mg/mL **PFC-1** , c) 5 mg/mL amorphous **TCPP-Co ligands,** and d) 5 mg/mL **PFC-72-Co** illuminated by a 635 nm red laser pointer.

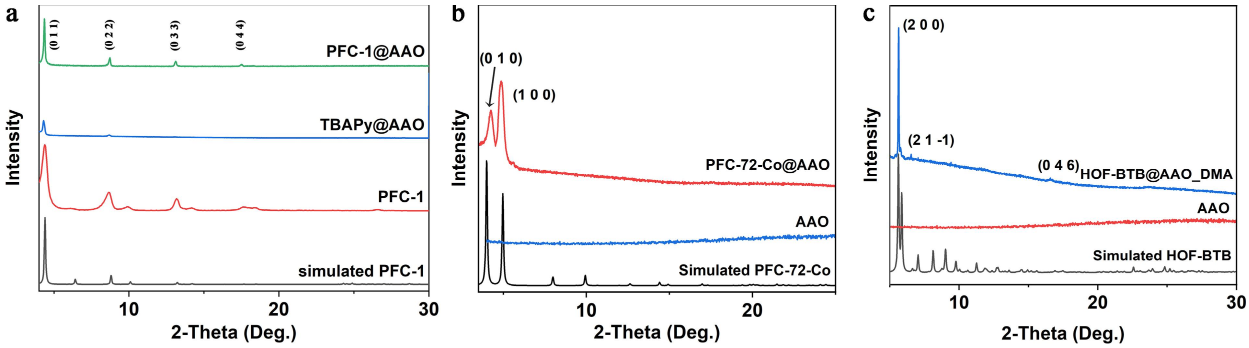

Supplementary Fig. 22. PXRD patterns of a) simulated PFC-1, as-synthesized powdery PFC-1, TBAPy@AAO membrane, and PFC-1@AAO membrane; b) simulated PFC-72-Co, as-synthesized powdery PFC-72-Co, and PFC-72-Co @AAO membrane; c) simulated HOF-BTB, AAO disk, and HOF-BTB@AAO_DMA membrane.

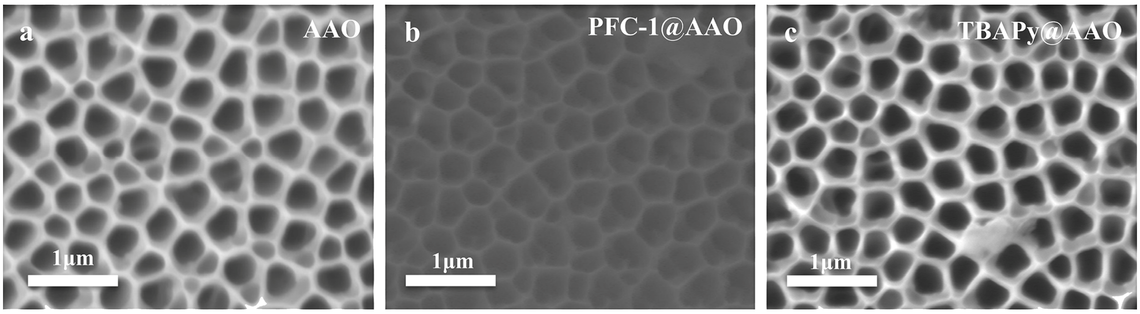

Supplementary Fig. 23. SEM images of a) **AAO disk**, b) **PFC-1@AAO membrane**, and c) **TBAPy@AAO membrane**.

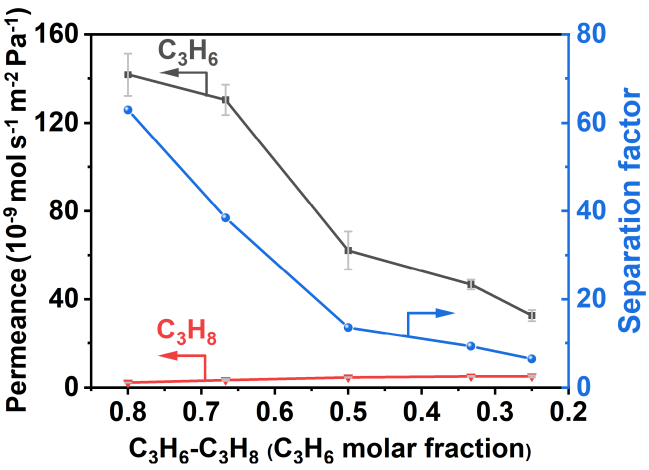

Supplementary Fig. 24. C_3H_6 partial pressure dependence of C_3H_6/C_3H_8 separation performance for **HOF-BTB@AAO membrane** at 27 °C and total pressure of 100 kPa. Error bars represent standard deviations.

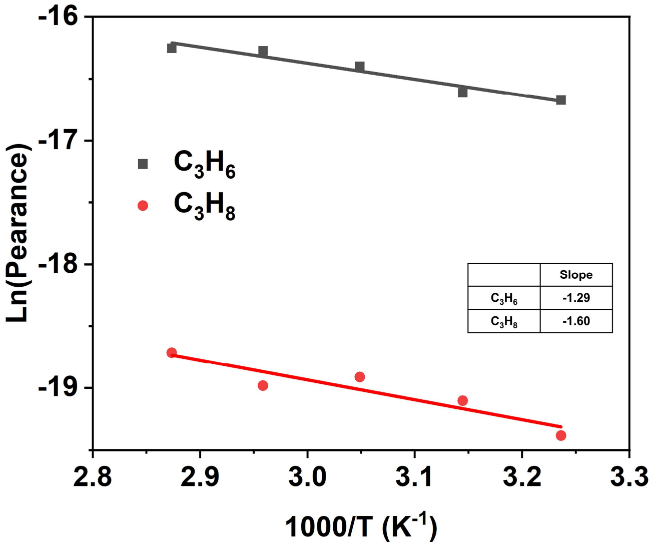

Supplementary Fig. 25. Arrhenius temperature dependence of C_3H_6 and C_3H_8 single gas permeance through the HOF-BTB@AAO membrane.

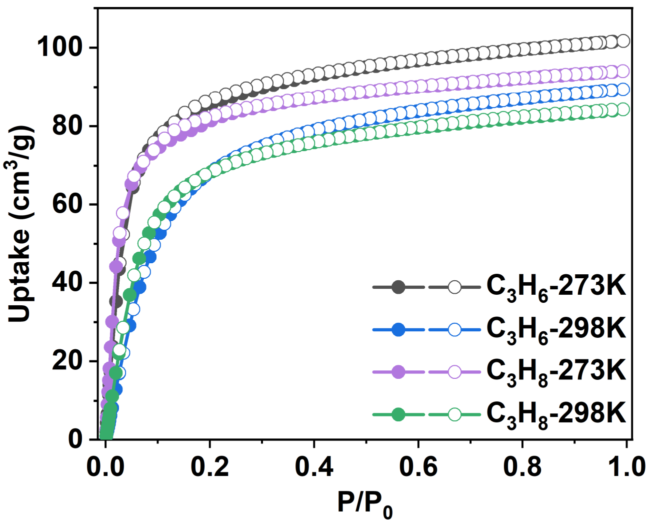

Supplementary Fig. 26. gas sorption isotherms of C_3H_6 and C_3H_8 for **HOF-BTB** at 273K and 298 K.

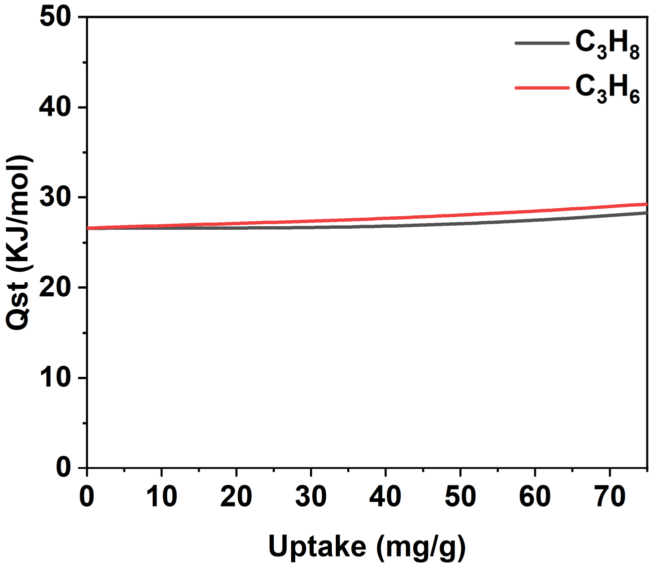

Supplementary Fig. 27. the heat of sorption of C_3H_6 (red) and C_3H_8 (black) for **HOF-BTB**.

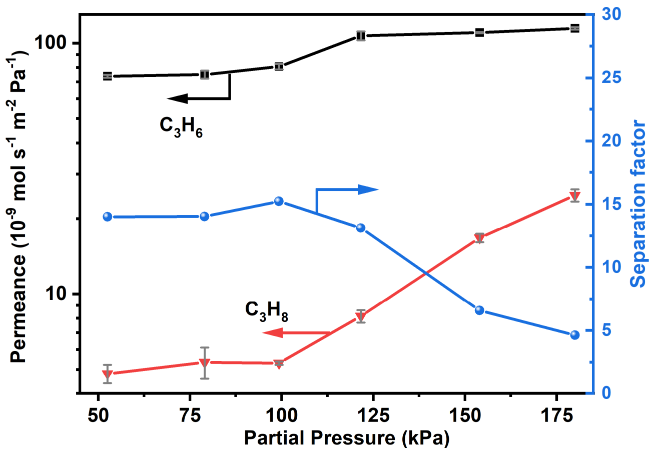

Supplementary Fig. 28. Pressure dependence of C_3H_6/C_3H_8 separation performance for **HOF-BTB@AAO membrane** at 27 °C with equimolar C_3H_6/C_3H_8 feed flow (single gas partial pressure as horizontal ordinate). Error bars represent standard deviations.

References

1 Stylianou, K. C. *et al.* A guest-responsive fluorescent 3D microporous metal-organic framework derived from a long-lifetime pyrene core. *Journal of the American Chemical Society* **132**, 4119-4130, doi:10.1021/ja906041f (2010).

2 Yin, Q. *et al.* An Ultra-Robust and Crystalline Redeemable Hydrogen-Bonded Organic Framework for Synergistic Chemo-Photodynamic Therapy. *Angew Chem Int Ed Engl* **57**, 7691-7696, doi:10.1002/anie.201800354 (2018).

3 Yin, Q. *et al.* Metallization-Prompted Robust Porphyrin-Based Hydrogen-Bonded Organic Frameworks for Photocatalytic CO(2) Reduction. *Angew Chem Int Ed Engl* **61**, e202115854, doi:10.1002/anie.202115854 (2022).

4 Zentner, C. A. *et al.* High surface area and Z' in a thermally stable 8-fold polycatenated hydrogen-

bonded framework. *Chemical Communications* **51**, 11642-11645, doi:10.1039/c5cc04219d (2015).
5 Hongwei, F. *et al.* COF–COF Bilayer Membranes for Highly Selective Gas Separation. *Journal of the American Chemical Society* **140**, 10094–10098, doi:doi:10.1021/jacs.8b05136 (2018).
6 Feng, S. *et al.* Fabrication of a Hydrogen-Bonded Organic Framework Membrane through Solution Processing for Pressure-Regulated Gas Separation. *Angewandte Chemie International Edition* **59**, 3840-3845, doi:10.1002/anie.201914548 (2020).
7 Wan, W., Sun, J., Su, J., Hovmoller, S. & Zou, X. Three-dimensional rotation electron diffraction: software RED for automated data collection and data processing. *Journal of Applied Crystallography* **46**, 1863-1873, doi:10.1107/S0021889813027714 (2013).
8 Wang, B. *et al.* A Porous Cobalt Tetraphosphonate Metal-Organic Framework: Accurate Structure and Guest Molecule Location Determined by Continuous-Rotation Electron Diffraction. *Chemistry - A European Journal* **24**, 17429-17433, doi:10.1002/chem.201804133 (2018).
9 Chen, P. *et al.* Crystalline Sponge Method by Three-Dimensional Electron Diffraction. *Frontiers in Molecular Biosciences* **8**, 821927, doi:10.3389/fmolb.2021.821927 (2021).
